# Increases in Heart Rate Variability Signal Improved Outcomes in Rapid Response Team Consultations: A Cohort Study

**DOI:** 10.1155/2018/1590217

**Published:** 2018-03-01

**Authors:** Nawal Salahuddin, Azam Shafquat, Qussay Marashly, Khaled Juan Zaza, Moh'd Sharshir, Moazzum Khurshid, Zeeshan Ali, Melissa Malgapo, Mouhamad Ghyath Jamil, Mohamed Shoukri, Mohammed Hijazi, Bandar Al-Ghamdi

**Affiliations:** ^1^Department of Critical Care Medicine, King Faisal Specialist Hospital & Research Centre, Riyadh 11211, Saudi Arabia; ^2^Section of Electrophysiology, Heart Center, King Faisal Specialist Hospital & Research Centre, Riyadh 11211, Saudi Arabia; ^3^College of Medicine, Alfaisal University, P.O. Box 50927, Riyadh 11533, Saudi Arabia; ^4^Department of Critical Care Medicine, king Faisal Specialist Hospital & Research Centre, Riyadh 11211, Saudi Arabia; ^5^Department of Nursing, King Faisal Specialist Hospital & Research Centre, Riyadh 11211, Saudi Arabia; ^6^National Biotechnology Center, King Faisal Specialist Hospital & Research Centre, Riyadh 11211, Saudi Arabia

## Abstract

**Background:**

Reduced heart rate variability (HRV) indicates dominance of the sympathetic system and a state of “physiologic stress.” We postulated that, in patients with critical illness, increases in HRV might signal successful resuscitation and improved prognosis.

**Methods:**

We carried out a prospective observational study of HRV on all patients referred to the rapid response team (RRT) and correlated with serial vital signs, lactate clearance, ICU admission, and mortality.

**Results:**

Ninety-one patients were studied. Significantly higher HRV was observed in patients who achieved physiological stability and did not need ICU admission: ASDNN 19 versus 34.5, *p*=0.032; rMSSD 13.5 versus 25, *p*=0.046; mean VLF 9.4 versus 17, *p*=0.021; mean LF 5.8 versus 12.4, *p*=0.018; and mean HF 4.7 versus 10.5, *p*=0.017. ROC curves confirmed the change in very low frequencies at 2 hours as a strong predictor for ICU admission with an AUC of 0.772 (95% CI 0.633, 0.911, *p*=0.001) and a cutoff value of −0.65 associated with a sensitivity of 78.6% and a specificity of 61%.

**Conclusions:**

Reduced HRV, specifically VLF, appears closely related to greater severity of critical illness, identifies unsuccessful resuscitation, and can be used to identify consultations that need early ICU admission.

## 1. Background

Extreme alterations in heart rate, blood pressure, and consciousness are parameters recognized as markers of “severe illness” and are used to mobilize rapid response teams or ICU consultations. RRT evaluation generally involves bedside assessment and resuscitation with eventual ICU admission if the patient does not stabilize. Prognostication in these patients is problematic. Early warning scores and, more recently, the quick Sequential Organ Failure Assessment (qSOFA) scores have been validated as strong predictors of outcome in septic patients and may be used to decide on early ICU transfer [1, 2]. Lactic acidosis develops with hypoperfusion (shock). The aim of resuscitation is to normalize perfusion, which can be measured by clearance of lactate [3]. However in cases of mitochondrial disorders (Type B lactic acidosis) or with failure of usual clearance routes (renal or hepatic failure), physicians cannot rely on lactic acid clearance as a goal of resuscitation.

Fluctuations of the R-R interval between consecutive heartbeats as well as the oscillations between consecutive, instantaneous heart rates are conventionally known as heart rate variability (HRV) and are accepted as an indicator of the dynamic equilibrium between sympathetic and parasympathetic divisions of the autonomic nervous system [4]. Time domain parameters measure HRV over a given period. These are calculated based on the time interval between successive normal sinus heart beats (NN interval which is expressed in milliseconds). The variability in these NN intervals can be expressed by several different parameters. SDNN refers to the standard deviation of the NN interval. SDANN is obtained by averaging NN intervals for each 5-minute segment and calculating its standard deviation. SDNN index or ASDNN is the average of the SDNN of each 5-minute segment over 24 hours. rMSSD (root mean square of successive differences) is calculated by squaring the difference in milliseconds between successive NN intervals, averaging it, and then taking its square root. pNN50 is the percentage of successive NN intervals that differ by more than 50 milliseconds (ms).

Heart rate variability follows cyclical patterns. Different physiological parameters can cause these cyclical changes albeit at different cycle lengths or frequencies. Oscillations in heart rate due to respiration, for example, occur in a rhythmic fashion over a cycle with frequency between 0.15 and 0.40 Hz (high frequency (HF)). Contribution of different factors affecting the heart rate variability can be calculated by analyzing the heart rate variability at very low frequency (VLF, 0.0033–0.04 Hz), low frequency (LF, 0.04–0.15 Hz), and high frequency (HF, 0.15–0.4 Hz).

Heart rate variability (HRV) has been described to indicate a balance between the sympathetic and parasympathetic nervous systems with *reduced* HRV, indicating dominance of the sympathetic system and a state of “physiologic stress.” In patients after myocardial infarction, reduced HRV is predictive of cardiac mortality [5] and sudden cardiac death [6]. Similarly, reduced HRV in septic patients presenting to the emergency department has been linked to higher mortality and greater likelihood of progression to shock [7–9] and, in ICU patients, with higher organ failure scores [10]. In patients surviving cardiac arrest, reduced HRV appears to be predictive of early mortality [11]. Heart rate variability is evaluated as both time domain and frequency domain measures [4, 12]. Time domain parameters estimate HRV over a 24-hour period. Frequency domain parameters can be measured hourly and as a mean over the monitoring period. Previous investigators have found that HRV measurements in both domains correlate with poor outcomes [7–9, 11].

Patients seen in RRT consultation need early markers that would indicate either stabilization or deterioration requiring ICU admission. HRV data may be useful in such a situation as it can reflect improvement or worsening of illness, but there is no literature addressing this particular population. We postulated that an increase in HRV with resuscitation may signal stabilization and may serve as a guide of clinical recovery along with the more conventional lactate and hemodynamic variables such as blood pressure and heart rate. The objectives of our study were to study HRV patterns in patients referred for critical illness and to determine if trends in HRV variables could identify patients not responding to resuscitation and therefore requiring ICU admission.

## 2. Methods

This was a prospective, observational study of consecutive rapid response team (RRT) consultations carried out from June 2015 to May 2016. Patients were evaluated and resuscitated by the RRT/ICU consultation teams as per usual routine. Adult patients without atrial or ventricular arrhythmias or previous pacemaker or internal cardiac defibrillator insertion were included in the study. Written consent was obtained for participation and monitoring. In addition to ongoing resuscitation, continuous EKG was recorded by a Holter monitor attached for 24 hours. Holter recordings were analyzed by using MARS Holter monitoring system (GE Healthcare) and proprietary software. All studies were manually scanned to ensure sinus rhythm, and that all abnormal beats were placed in appropriate bins. Recordings with atrial fibrillation were excluded from the analysis. Heart rate variability analysis was according to guidelines established by the Task Force of the European Society of Cardiology and the North American Society of Pacing and Electrophysiology [4]. Heart rate variability was measured as time domains measured over 24 hours (SDNN, ASDNN, rMSSD, pNN50%, SDANN, and mean NN) and frequency domains measured hourly (very low frequency (VLF), low frequency (LF), high frequency (HF), and low/high ratio) as well as a mean value taken over the 24-hour monitoring period. Changes from baseline were calculated. Frequency domains were reported as power in ms^2^. As per institution-approved criteria, patient improvement and stabilization was defined by normalization or ≥10% reduction in serum lactate levels, ≥15% reduction in heart rate, or increase in systolic blood pressure over the first few hours of resuscitation or weaning off vasopressors and clinical judgment. These patients would not routinely be admitted to the ICU. Patients not improving were admitted to the ICU as decided by the treating RRT/ICU consulting physician. Holter monitoring was continued in all patients for the prespecified 24-hour period. Patients could be treated at the bedside by the RRT using institution-approved therapeutic interventions that include dopamine up to a dose of 5 mcg/kg/minute administered through a peripheral line, any crystalloid fluids or 5% albumin boluses, noninvasive ventilation by face mask, antibiotics, furosemide, endotracheal intubation, and emergency medications from the crash cart (epinephrine, atropine, naloxone, fentanyl, and 20% glucose). All patients were followed for 72 hours after enrollment with serial measurements of physiological data, biochemical data, and for outcomes (ICU admission and mortality) until day 28 from enrollment. HRV variables were collected both before and after ICU admission. Our study was designed to assess if measurement of HRV and change from baseline could assist clinicians in prognostication. However, as it was not known how soon during resuscitation a change may be seen, we measured hourly HRV for a full 24-hour period regardless of whether the patient was or was not admitted to the ICU.

### 2.1. Statistical Analysis

Data are reported as means (SD) or medians (IQR 25%–75%) for skewed variables. Continuous variables were compared using Student's *t*-test or Mann–Whitney *U* test; categorical variables were compared using the chi-square or Fisher's exact test where appropriate. Stepwise univariate and multivariate logistic regression was used to determine independent predictors of 28-day mortality and ICU admission. Multiple comparisons using ANOVA were used to test whether the VLF values were significantly different over time. Receiver-operating characteristic (ROC) curves were constructed to identify a cutoff value of the change in VLF at 4 and 6 hours with the highest predictive abilities (sensitivity and specificity) for ICU admission. Two-sided *p* values < 0.05 were used to determine statistical significance. SPSS version 22.0 was used for analysis.

## 3. Results

Ninety-six patients were enrolled during the study period; five were excluded for atrial fibrillation or errors with Holter recordings. Mean age was 49.9 ± 22.3 years, 54.9% (50 patients) were male, mean APACHE II score was 23.5 ± 7.3, and mean day 1 SOFA score was 9.1 ± 4.9. Diagnoses at hospitalization included 9 liver cirrhosis patients (10%), 8 chronic respiratory disease patients (9%), 8 renal disease patients (9%), 29 malignancy cases (32%), 11 chronic multiorgan dysfunction cases (12%), and 12 other cases (HIV, tuberculosis, lupus, elective surgery, and pregnancy-related) (13%). Diagnoses at RRT consultation are shown in Table 1. RRT consultations were 52.7% from medicine (48 patients), 29.7% from hematology/oncology (27 patients), 15.4% from surgery (14 patients), and 2.2% from obstetrics/gynecology (2 patients).

RRT interventions administered included the following: 58 patients (64%) received antibiotics, 49 patients (54%) were given fluid boluses that included a mixture of both isotonic crystalloids and 5% albumin, 2 (2%) were given both fluids and started on dopamine infusions titrated to a MAP of 65 mmHg and higher or to a maximum dose of 5 mcg/kg/minute, 8 (9%) patients received furosemide as bolus doses, 4 (4%) were given naloxone, 10 (11%) were started on noninvasive ventilation, and 15 (16.5%) received both noninvasive ventilation and furosemide.

Seventy-seven patients (84.6%) were admitted to the ICU, and mean time from RRT consultation to ICU admission was 3.6 ± 2.3 hours (range 1–12). ICU mortality was 32.4% (25 of 77 patients) and 28-day mortality was 28.6% (26 patients of 91).

Heart rate variability amongst the entire patient cohort was SDANN/ms 56 (37.7–91.5), ASDNN/ms 20 (13.7–35.2), rMSSD/ms 14.5 (10–30.7), pNN50% 1.3 (0.2–8.9), mean NN/ms 659.5 (547.7–750.5), and SDNN/ms 67 (46.7–100) for the time domains. For the frequency domains, the values were as follows: 10.8 (5.8–17.2) for the very low frequency (VLF) (ms^2^), 6.4 (3.4–12.9) for low frequency (LF) (ms^2^), 5.2 (3.3–11.2) for high frequency (HF) (ms^2^), and 1.09 (0.8–1.3) for the low/high ratio (L/H). Vasopressor use appeared to have no impact on HRV, and *p* is nonsignificant for both time and frequency domains (Table 2).

**Figure 1:**
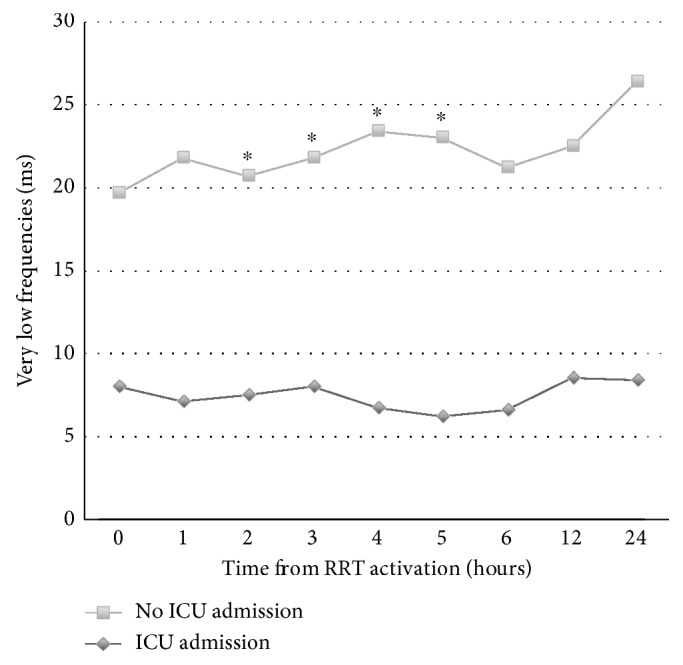
Trajectories of very low frequency domains between patients requiring and not requiring ICU admission. ^∗^Significant differences with *p* values < 0.05.

HRV was significantly higher in patients who did not need ICU admission. These patients also showed significantly greater hourly improvements in VLF during the resuscitative period. A significant divergence was identified in the VLF as early as 2 hours into resuscitation (Table 3 and Figure 1).

Significant correlations were seen between lactate clearance at 24 hours and changes in VLF at 2 hours (*r*^2^=−0.234,  *p*=0.025) and at 3 hours (*r*^2^=−0.232,  *p*=0.027). Significant correlations were also observed between change in heart rate at 12 hours and VLF change at 3 hours (*r*^2^=−0.23,  *p*=0.025), at 4 hours (*r*^2^=−0.288,  *p*=0.006), at 5 hours (*r*^2^=−0.241,  *p*=0.021), and at 6 hours (*r*^2^=−0.26,  *p*=0.013).

Univariate regression for indicators of ICU admission identified baseline SOFA score (odds ratio (OR) 12, *p*=0.021), change in VLF at 2 hours (OR 0.89, *p*=0.03), at 3 hours (OR 0.89, *p*=0.038), at 4 hours (OR 0.86, *p*=0.003), at 5 hours (OR 0.89, *p*=0.11), and at 6 hours (OR 0.89, *p*=0.022); mean arterial pressure (MAP) at 3 hours (OR 1.08, *p*=0.025); changes in L/H ratio at 1 hour (OR 0.21, *p*=0.07) and at 4 hours (OR 0.21, *p*=0.02); and VLF at 4 hours (OR 0.95, *p*=0.022) and at 5 hours (OR 0.96, *p*=0.04). On multivariate regression, change in VLF at 2 hours (OR 0.68, 95% CI 0.52, 0.90, *p*=0.007), change in VLF at 4 hours (OR 0.72, 95% CI 0.54, 0.96, *p*=0.026), change in VLF at 6 hours (OR 0.58, 95% CI 0.38, 0.88, *p*=0.011), MAP at 3 hours (OR 1.3, 95% CI 1.05, 1.61, *p*=0.015), and baseline SOFA score (OR 1.77, 95% CI 1.11, 2.8, *p*=0.015) remained significant.

**Figure 2:**
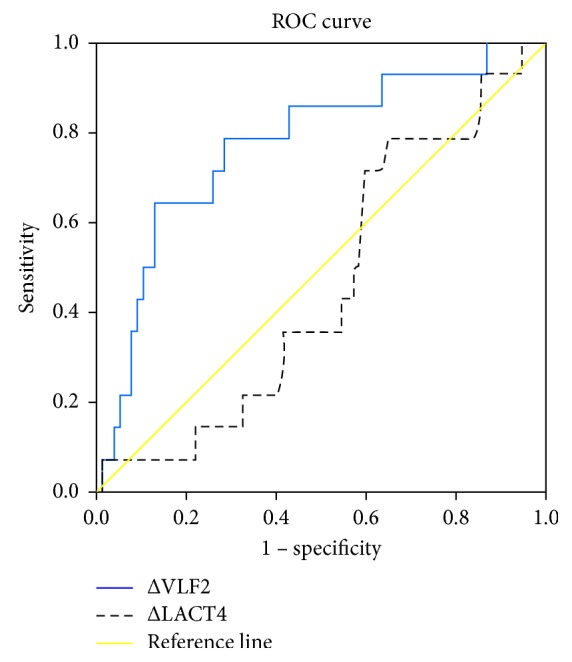
Receiver-operating characteristic curve identifying changes in VLF at 2 hours (AUC 0.772, 95% CI 0.633, 0.911, *p*=0.001) from baseline as a significant indicator of ICU admission. Change in serum lactate at 4 hours (AUC 0.459, 95% CI 0.31, 0.60, *p*=0.62) is shown as a comparison.

Receiver-operating characteristic curves confirmed the change in VLF at 2 hours as a strong indicator of ICU admission with an AUC of 0.772 (95% CI 0.633, 0.911, *p*=0.001) with a cutoff value of −0.65 associated with a sensitivity of 78.6% and specificity of 61%. A comparative lactate clearance was not useful (AUC 0.459, 95% CI 0.31, 0.60, *p*=0.62) (Figure 2).

Patients who survived the ICU admission had significantly lower APACHE II and SOFA scores with significantly greater improvements in serum lactate. HRV was significantly higher (mean VLF 5.5 (4.2, 11.7) versus 11.9 (8.3, 18.7), *p*=0.002; rMSSD 16.5 (9–20) versus 25.2 (12–35) *p*=0.017; mean NN/ms 581.5 (513–662) versus 714 (589–792) *p* ≤ 0.001; L/H at 4 hours 0.8 (0.5, 1.1) versus 1.2 (0.7, 1.6) *p*=0.014) in survivors and exhibited greater improvements over follow-up (VLF change at 4 hours −0.5 (−3.6, −0.2) versus 0.09 (−1.5, 4.9) *p*=0.004).

**Figure 3:**
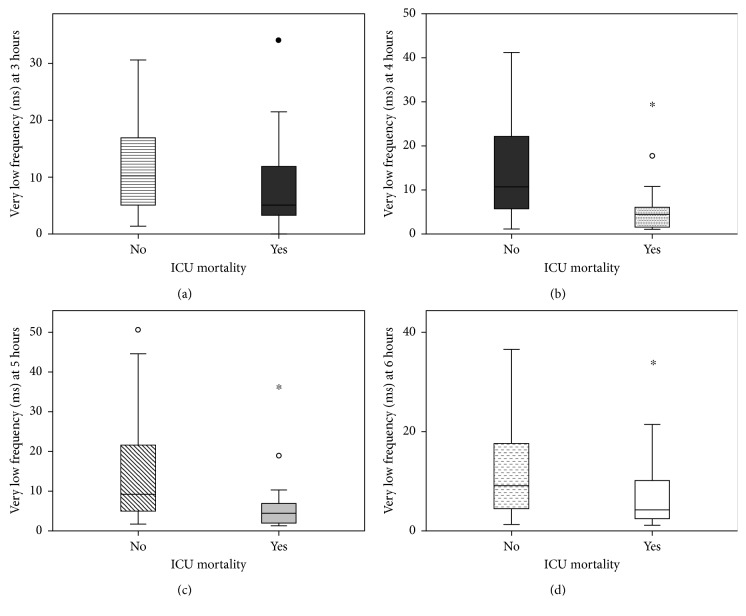
Trends in hourly very low frequencies between ICU survivors and nonsurvivors.

Patients surviving to 28 days had significantly higher HRV; time domains (ASDNN 14 versus 24, *p*=0.012; rMSSD 13 versus 19, *p*=0.037; mean NN 581 versus 685, *p*=0.004) and frequency domains (mean VLF 5.7 versus 11.7, *p*=0.005; mean LF 4.6 versus 7.5, *p*=0.038) had significantly lower SOFA scores, heart rates, serum lactate levels, and greater lactate clearances at 12 and 24 hours. No significant differences were found in lactate clearance at 4 and 6 hours and in baseline mean arterial pressure between survivors and nonsurvivors (Figure 3).

## 4. Discussion

In this study, we demonstrate that heart rate variability has clinical utility in the assessment and resuscitation of critically ill patients. Patients in whom the critical illness stabilized tended to have a higher heart rate variability and showed greater hour-by-hour increases, compared to those who failed to improve and had to be admitted to the ICU. Survival differences were also predictable by mean and hourly heart rate variability.

Though our study is amongst the first to look at HRV trajectories in RRT consultations, low HRV has been previously described as a marker of greater illness and worse outcomes. In 1994, Tsuji et al. [13] reported that, of the 736 original subjects in the Framingham Heart Study, analysis of the first 2 hours of ambulatory ECG revealed a significant association between all-cause mortality and the very low frequency (*p* < 0.0001), low frequency (*p* < 0.0001), high frequency (*p*=0.0014), total power (*p* < 0.0001), and the SDNN (*p*=0.0019). This was followed by reports of associations between low HRV and sudden cardiac death [14], stroke outcomes [15, 16], prognosis in heart failure [17] and risk of cardiac arrest [18].

Amongst the ICU population, Schmidt et al. [19], in an observational study of 90 patients with 24-hour ECG monitoring, described significantly reduced HRV in patients with multiorgan failure and lnVLF as an independent predictor of 28-day mortality (AUC 0.68, 95% CI 0.55, 0.8). Papaioannou et al. [20] measured HRV as variance (exponent alpha2) and approximate entropy (ApEn) by analyzing daily heart rates recorded from bedside monitors. They described lower ApEn in nonsurvivors compared to survivors (0.53 ± 0.25 versus 0.62 ± 0.23, *p*=0.04) and higher variance and ApEn in patients with low SOFA scores (0.47 ±0.51 versus 0.10 ± 0.65, *p* < 0.001; 0.67 ± 0.28 versus 0.49 ±0.24, *p* < 0.001). In 2016, Bishop and coworkers [21] compared HRV with APACHE II scoring in 55 ICU patients. They described a robust independent predictive ability for 30-day mortality with OR 0.6 and 95% CI 0.396, 0.911. Similar to these reports, we have also demonstrated that reduced HRV is associated with lower ICU and 28-day survival.

The VLF band falls between 0.0033 and 0.04 Hertz in the HRV spectrum. Kember et al. [22, 23] demonstrated that the VLF band is generated by the heart's intrinsic nervous system and is modulated by efferent sympathetic activity. It is postulated that the activity of the autonomic nervous system, especially regulation of the renin-angiotensin system and thermoregulation, may contribute to this HRV band [24]. The VLF band has been described to reflect a high inflammatory state [25, 26] and has specifically been described to have the highest association with adverse outcomes [13, 17, 19, 21]. Therefore, the VLF band should be considered an intrinsic rhythm necessary to health and well-being. In our study, we also found the VLF band to be strongly associated with both unsuccessful resuscitation and increased mortalities. Additionally, we found the VLF to have predictive ability above that of lactate clearance.

The strengths of our study are that our study population was a diverse group similar to most populations that conventional RRT consultation would comprise. We were also able to demonstrate that vasopressor use had no effect on HRV. Limitations include the inability to generalize our results to patients with pacemakers or atrial arrhythmias.

We set out to demonstrate was that, in patients who met criteria for RRT consultation (and of whom 85% were deemed sick enough to require ICU admission), HRV variables showed significant initial differences and diversion between patients who stabilized with minimal resuscitation by the RRT team and did not require ICU admission compared to those who were admitted to the ICU. Since most patients were admitted within a mean period of 3.6 ± 2.3 hours (range 1–12), we also assessed HRV variables as predictors of 28-day survival, that is, whether the trajectories of HRV variables were an indicator of response. We confirmed this by observing significantly different trajectories of change in HRV variables between survivors and those who died. We were also able to identify a cutoff value of the VLF variable that separated these two groups. Through our observation of the differences in HRV variables amongst RRT consults requiring ICU admission and in 28-day survival of the entire study cohort, we surmise that, in the future, these variables and the cutoff values maybe helpful to clinicians to predict outcomes. We have shown that “real-world” heart rate variability monitoring is a practical tool that can be used to assess the adequacy of resuscitation and improvement in short-term hourly intervals and allows for rapid assessment in RRT/ICU consultations. The growing availability of smart phone applications that measure HRV may allow RRT physicians to perform bedside HRV monitoring. Certainly, validation of these applications with comparison to EKG recordings appears to be the next step.

## 5. Conclusions

Reduced HRV, specifically VLF, appears closely related to greater severity of critical illness, identifies unsuccessful resuscitation, and can be used to identify consultations that need early ICU admission. Based on our results, prognostication using real-time HRV assessment at the bedside is a promising next step.

## Figures and Tables

**Table 1:** Baseline patient characteristics.

	Number
Sepsis	58 (64%)
APACHE II score	23.5 ± 7.3
*Diagnosis at RRT activation*	
Acute respiratory failure^3^	30 (33%)
Hepatic and GI failure^4^	19 (21%)
Renal failure^5^	15 (16.5%)
Malignancy	20 (22%)
Miscellaneous^6^	7 (8%)
*Reason for RRT consultation* ^7^	
Tachypnea	50 (55%)
Hypotension	39 (43%)
Brady or tachycardia	32 (35%)
Depressed mentation	23 (25%)
Subjective concern of nurse	3 (3%)
*SOFA* ^1^ *scores*	
Day 0	9.1 ± 4.9
Day 1	8.8 ± 5.1
Day 2	7.9 ± 5.3
Serum lactate at baseline (mmol/L)	1.8 (IQR 1.1–4.6)
MAP^2^ at baseline (mmHg)	78 ± 18.9
*Vasopressors*	
On day 0	36 (40%)
On day 1	34 (37%)
On day 2	19 (21%)

^1^SOFA, Sequential Organ Failure Assessment score; ^2^MAP, mean arterial pressure; ^3^acute respiratory failure includes ARDS; ^4^decompensated cirrhosis, cholangitis, pancreatitis, and GI bleed; ^5^acute kidney injury (end-stage renal disease); ^6^intracranial hemorrhage, stroke, encephalitis, and collagen vascular diseases; ^7^more than one.

**Table 2:** Comparisons of heart rate variability domains between patients by vasopressor use.

	No vasopressors (*n*=53)	Vasopressors (*n*=35)	*p* value
VLF (ms^2^) (over 24 hours)	10.8 (6–15.7)	10.4 (5.3–21.7)	ns
LF (ms^2^) (over 24 hours)	6.3 (3.5–11.4)	8.7 (3.4–17.5)	ns
HF (ms^2^) (over 24 hours)	4.8 (3–8.9)	8.4 |(3.3–15.2)	ns
L/H ratio (over 24 hours)	1 (0.8–1.4)	1.1 (0.8–1.3)	ns
SDANN/ms	52.5 (35–77.7)	71 (40.7–107.7)	ns
ASDNN/ms	19 (13.5–31.2)	26 (13.2–43)	ns
rMSSD/ms	14 (10.7–24.2)	24.5 (10–35)	ns
pNN50%	0.95 (0.07–6)	5 (0.4–10.5)	ns
Mean NN/ms	648.5 (546.5–735.2)	672 (550–811.5)	ns
SDNN/ms	60 (45.5–94.5)	78.5 (48–112.7)	ns

Data are shown as mean ± standard deviations, mean (range), and median (25–75 interquartile range) as appropriate. VLF, very low frequency; LF, low frequency; L/H, low/high ratio; HF, high frequency.

**Table 3:** Comparisons between RRT consultations who did or did not need ICU admission.

	Required ICU admission (77)	Not admitted to ICU (14)	*p* value
*SOFA scores*			
Day 0	9.6 ± 5.1	6 ± 2.3	<0.001
Day 1	9.7 ± 5	3.8 ± 2.5	<0.001
Day 2	8.7 ± 5.3	3.2 ± 2.2	<0.001
MAP at 3 hours	70 ± 6.2	81.4 ± 15.6	<0.001
MAP at 4 hours	73.1 ± 13	82.5 ± 15.3	0.047
Lactate at 1 hour (mmol/L)	2.7 (0.1, 16.7)	1.3 (0.8, 2.1)	0.001
Lactate at 12 hours (mmol/L)	2.5 (0.1, 17.9)	1.7 (0.5, 2.4)	0.024
Lactate at 24 hours (mmol/L)	2.5 (0.1, 19.7)	1.4 (0.6, 2.1)	0.01
*Heart rate variability domains*			
ASDNN/ms	19 (12, 31.7)	34.5 (19.5, 41.5)	0.032
rMSSD/ms	13.5 (10, 31.7)	13.5 (10, 31.7)	0.046
Mean VLF (ms^2^)	9.4 (5.3, 15.2)	17 (11.3, 21.7)	0.021
Mean LF (ms^2^)	5.8 (3.2, 11.2)	12.4 (7.5, 17.4)	0.018
Mean HF (ms^2^)	4.7 (2.8, 10.4)	10.5 (7.5, 17.4)	0.017
*Hourly differences in frequency domains*			
*Very low frequency in ms*^*2*^			
VLF at 2 hours	7.5 (4.2, 14.8)	13.2 (6.8, 19.7)	0.035
VLF at 3 hours	8 (3.9, 14.5)	13.8 (8.6, 25)	0.025
VLF at 4 hours	6.7 (4, 18.5)	16.7 (10.5, 24.7)	0.004
VLF at 5 hours	6.2 (3.2, 12.8)	16.8 (10.7, 24.2)	0.003
VLF at 6 hours	6.6 (3.6, 13.4)	14.6 (10.1, 21.3)	0.006
VLF at 24 hours	8.4 (3.6, 13.7)	18 (8, 25.7)	0.025
Change in^∗^ VLF at 1 hour	−0.4 (−1, −1.1)	1.9 (−0.5, −3.8)	0.027
Change in VLF at 2 hours	−1 (−3.3, 0.8)	2.4 (0.1, 4.7)	0.001
Change in VLF at 3 hours	−0.9 (−2.1, 1.8)	3.3 (−1.2, 9.7)	0.025
Change in VLF at 4 hours	−0.4 (−2.7, 2.2)	5.2 (−0.3, 11)	0.003
Change in VLF at 5 hours	−0.5 (−2.2, 1.6)	6.5 (−0.8, 8.2)	0.005
Change in VLF at 6 hours	−0.2 (−2.2, 1.3)	3.7 (0.5, 8)	0.003
Change in LH at 1 hour	0.002 (−0.1, 0.07)	0.1 (0.001, 0.2)	0.004
Change in LH at 4 hours	0 (−0.2, 0.2)	0.2 (−0.09, 0.6)	0.034
Change in LH at 5 hours	2.8 (1, 10.5)	9.6 (3, 15.3)	0.043
Change in LH at 24 hours	0 (−0.4, −0.3)	0.2 (−0.02, 0.6)	0.028
Change in LF at 1 hour	−0.1 (−1.1, −0.9)	1.4 (0.04, −5.6)	0.015
Change in LF at 2 hours	−0.5 (−2.2, 0.6)	1.9 (0.5, 3.5)	<0.001
Change in LF at 6 hours	0.13 (−1.7, −0.8)	2.7 (0.5, 6.4)	0.002

Data are shown as mean ± standard deviations, mean (range), and median (25–75 interquartile range) as appropriate. ^∗^“Change in” refers to change from baseline. MAP, mean arterial pressure; VLF, very low frequency; LF, low frequency, L/H, low/high ratio; HF, high frequency.

## Abbreviations

HRV: Heart rate variability
RRT: Rapid response team
SOFA score: Sequential Organ Failure Assessment score
qSOFA score: Quick Sequential Organ Failure Assessment score
SDNN: Standard deviation of NN intervals
SDANN: Standard deviation of the average NN intervals
rMSSD: Root mean square of successive differences
pNN50%: Proportion of NN50 divided by total number of NNs
VLF: Very low frequency
LF: Low frequency
HF: High frequency
L/H: Low/high ratio
AUC: Area under the curve
ROC: Receiver-operating characteristic
OR: Odds ratio
CI: Confidence intervals.
